# Development of 17-4 PH Stainless Steel for Low-Power Selective Laser Sintering

**DOI:** 10.3390/ma18020447

**Published:** 2025-01-19

**Authors:** Yu-Deh Chao, Shu-Cheng Liu, Fu-Lin Chen, Mayur Jiyalal Prajapati, Ajeet Kumar, Jung-Ting Tsai, Jeng-Ywan Jeng

**Affiliations:** 1 High Speed 3D Printing Research Center, National Taiwan University of Science and Technology, No. 43, Sec. 4, Keelung Rd., Taipei 106, Taiwan; hjkhjkhjk86@yahoo.com.tw (Y.-D.C.); d11003003@mail.ntust.edu.tw (S.-C.L.); ww850149@gmail.com (F.-L.C.); mayurprajapati@mail.ntust.edu.tw (M.J.P.); tsaij@mail.ntust.edu.tw (J.-T.T.); 2 Department of Mechanical Engineering, National Taiwan University of Science and Technology, No. 43, Sec. 4, Keelung Rd., Taipei 106, Taiwan; 3 Department of Design, Indian Institute of Technology Guwahati, Guwahati 781039, India; ajeetkumar@iitg.ac.in; 4 School of Aerospace, Mechanical and Mechatronics Engineering, The University of Sydney, Camperdown, NSW 2006, Australia

**Keywords:** 17-4 PH stainless powder, selective laser sintering, shrinkage analysis, tensile strength

## Abstract

Selective laser sintering (SLS) is one of the prominent methods of polymer additive manufacturing (AM). A low-power laser source is used to directly melt and sinter polymer material into the desired shape. This study focuses on the utilization of the low-power laser SLS system to successfully manufacture metallic components through the development of a metal–polymer composite material. In this study, 17-4 PH stainless powders are used and mixed with polyoxymethylene (POM) and high-density polyethylene (HDPE) to prepare the composite powder material. The polymeric mixture is removed during the thermal degreasing process and subsequent sintering results in a solid metallic component. Sinterit Lisa with a 5 W, 808 nm laser source is used to fabricate the green part. For the printing parameters of 140 °C, laser power of 35.87 mJ/mm^2^, and layer thickness of 100 μm, the printed samples achieved a maximum density of 3.61 g/cm^3^ and a complete shape. After sintering at 1310 °C for 180 min, the tensile strength of the shrunk sample is 605.64 MPa, the hardness is HRC 14.8, the average shrinkage rate is 22%, and the density is 7.57 g/cm^3^, which can reach 97% of the theoretical density. This process allows the use of a wide range of particle sizes that the usual AM technologies have, making it a low-cost, low-energy-consumption, high-speed AM technology.

## 1. Introduction

Powder Bed Fusion (PBF) is a family of additive manufacturing (AM) processes that involves the fusion of powdered materials layer by layer using a focused energy source. The usage of powdered materials offers high precision in creating complex geometries along with excellent mechanical properties [1,2]. Selective laser sintering (SLS) is a subset of PBF technology where the powdered materials are fused using the laser beam to create complex multifunctional geometries [3,4]. SLS has been predominantly used to produce prototypes and end-use components using thermoplastic polymeric materials such as nylon, polyester, polyvinylchloride (PVC), acrylonitrile butadiene styrene (ABS), etc., due to the usage of low-power laser energy sources [5,6,7]. To fabricate metallic components using a moderately low-powered laser, two or more metal powders are mixed having different melting points such that the high melting point powder becomes the structural material whereas the low melting point powder becomes the binder. This process is known as Direct Metal Laser Sintering (DMLS) [8]. DMLS offers several advantages such as the ability to produce accurate and precise components with a shorter production cycle. It also reduces post-processing operations significantly due to the absence of binder materials. However, the drawbacks of the DMLS process have severely restricted its use. Issues such as balling, high residual stress and porosity, microstructural defects, and non-homogeneous temperature distribution make it a difficult process to control [9].

Instead of mixing different metallic materials such as the DMLS process, metallic components can be produced in an indirect way using a low-cost SLS process by mixing polymers with metal powders. One of the simplest methods is the mechanical mixing of metal powders with polymer binder powders, but due to the density variation, powders become segregated, which results in poor binder efficiency. The other method is to coat the metal powders with polymer binders. The coated powders result in low binder content with higher binder efficiency. The common adhesive coating method has five steps: Step 1 is material weighing, Step 2 is mixing metal powder with a specific adhesive, Step 3 is adding a specific solvent, Step 4 is heating and stirring at a specific temperature until the adhesive is completely dissolved in the solvent, and Step 5 is vacuuming to remove the solvent, at which point the adhesive is coated on the outside of the powder [10]. The polymer binders for the indirect SLS process are mainly amorphous polymers such as poly (methyl methacrylate) (PMMA), PMMA-co-n-butyl methacrylate (BMA), and epoxy. The green parts must show sufficient mechanical properties to retain the desired shape and dimensions during handling and post-processing. One way to increase green part strength would be simply to increase the amounts of polymer binders. However, as the binders are removed by thermal processes, void spaces are left behind. High contents of polymer binders result in relatively larger amounts of void spaces upon high-temperature sintering, which can lead to unacceptable amounts of shrinkage in the finished part. Another problem with incorporating high-content polymer binders requires longer annealing times to remove the binder, which reduces efficiency and adds costs. Selective Laser Melting (SLM) and Directed Energy Deposition (DED) devices require high-power lasers to melt and shape metal powders [11,12]. When using metal powder printing, powder materials with a smaller particle size range are required, which is expensive and used in vacuum or inert gas environments, resulting in high production costs. Binder Jetting (BJ) technology can shorten printing time, but the post-processing time of this technology is relatively long [13]. Due to the melting temperature of polymer materials being much lower than that of common metals, printing with polymer materials is easier than printing with metal materials, and the energy of 3D printing equipment also increases with the melting point of the material.

The present study focuses on countering such issues by developing a novel coating on 17-4PH steel which is an iron alloy composed of copper (Cu), niobium (Nb), and chromium (Cr). A low-wattage laser provides energy to the composite powder, melting the polymer portion of the powder to form the object. This method allows the manufacturing of metal parts using lower-cost SLS equipment. The focus is on the precipitation-hardening properties of this steel, which belongs to the martensite phase in the stainless steel classification. Interestingly, the hardening of this stainless steel does not rely on carbon content to obtain the martensite phase, but rather copper ions precipitate at low temperatures to cause lattice distortion and obtain this phase. In this study, a composite material powder suitable for SLS equipment printing is developed. The metal powder is coated and dispersed with a polymer binder, and its structure is one type of core–shell structure. The particle size range of the metal powder used is between 1 and 60 microns, which is widely distributed in the MIM process, rather than the narrow particle size distribution between 40 and 60 microns commonly used in metal 3D printing.

The main advantages of this powder are high-temperature resistance, corrosion resistance, high strength and hardness, weldability, and grindability. The application areas include aerospace, petrochemicals and chemicals, ocean engineering, medical devices, electronic communication, and wearable devices.

## 2. Materials and Methods

### 2.1. Material Selection

**Figure 1:**
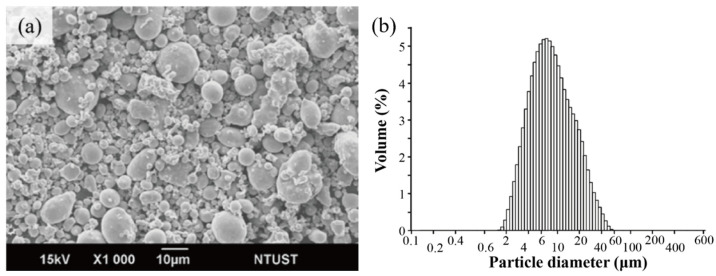
(**a**) 17-4PH water-atomized powder; (**b**) particle size analysis.

The metallic material used in this study is water-atomized 17-4 PH martensitic precipitation-hardened stainless steel procured from Tiz-Advanced Alloy Technology CO., Ltd. China, Quanzhou City, Fujian Province. Figure 1a shows the powder morphology and particle size distribution of the selected material. The shape of the powder shown in the figure is non-spherical, with a rounded surface morphology and slight agglomeration (distributed by Jiahui Optoelectronics). Its chemical composition meets the Metal Powder Industries Federation (MPIF 35) standard. The particle size distribution of the original 17-4PH powder was confirmed using a Beckman Coulter LS230 laser particle size analyzer (Brea, CA, USA), with a D50 of 8.23 μm (Figure 1b).

The adhesive components used in this study include the main component polyoxymethylene (POM) as a filler. It is catalytically decomposed into formaldehyde (CH_2_O) gas by oxalic acid gas and is removed during the degreasing process in the later stage. High-density polyethylene (HDPE) is a secondary component that acts as a high-temperature conformal agent and is removed during the sintering process, and stearic acid (SA) is a surfactant. The properties of the adhesive components are shown in Table 1.

### 2.2. Experimental Methods

A Sinterit Lisa SLS 3D printer from Krakow, Poland, for additive manufacturing, with a laser power of 5 W and a wavelength of 808 nm is used for printing the samples. To shape the 17-4PH powder, a core–shell material structure was designed to encapsulate the metal powder inside a polymer shell [14]. During the powder fabrication process, only a portion of the polymer needs to be melted and bonded.

**Figure 2:**
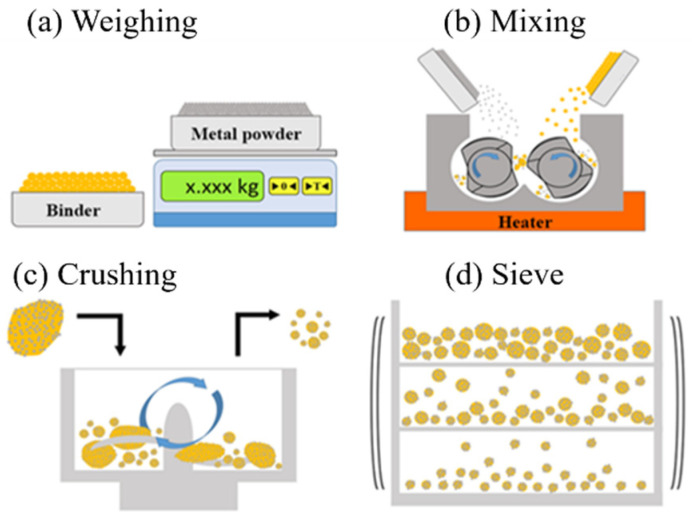
Metal powder and binder mixing process for forming the composite powder.

**Figure 3:**
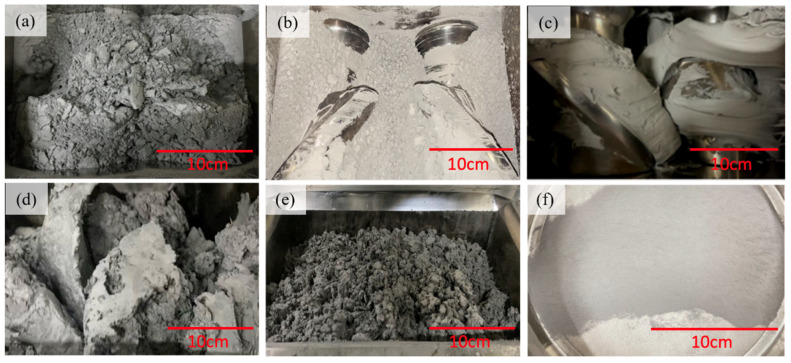
Process of adhesive coating 17-4PH powder: (**a**) removal of moisture from 17-4PH powder; (**b**) addition of binders (POM, HDPE, SA); and (**c**) mixing of the metal powder with binders to form a dough-like texture. (**d**) Block-shaped composite materials. (**e**) Composite materials are in granular form. (**f**) The composite material is in the form of gravel.

Figure 2 shows the schematic representation process used in this study to produce composite powders, which includes heating the metal powder and binder material, and mixing them under pressure using a rotor. When the binder is in a molten state, the metal powder is uniformly dispersed and mixed with the binder to form a hybrid composite material. After cooling, the material is crushed and sieved to obtain a composite powder. Figure 3 shows the manufacturing process of mixing metal powder and polymer binder using a mixer, which is divided into six stages. In the first stage, the metal powder is placed in a mixing chamber and heated to 160 °C to remove moisture from the powder, as shown in Figure 3a. In the second stage, after adding binders (POM, HDPE, and SA), it is heated to 170 °C as shown in Figure 3. In the third stage, the adhesive is completely melted and forms a dough shape. At this point, the metal powder and adhesive are fully mixed as shown in Figure 3c. In the fourth stage, the heater is turned off to start cooling the composite material, and it can be observed that the composite material begins to harden and form blocks as shown in Figure 3d. In the fifth stage, the temperature of the composite material continues to decrease until it becomes gravel-like as shown in Figure 3e. After powdering the gravel-like composite material particles, the LS-300T (Lao Song Machinery Co., Ltd., New Taipei, Taiwan) vibrating screen was used for screening as shown in Figure 3f. The vibrating screen has 120, 140, 170, 200, 230, 270, and 325 mesh screens, and the particle size distribution range of the screens is used to investigate the effect on printing parameters.

The Coulter LS 230 (Brea, CA, USA) particle size analyzer from Beckman Coulter, Inc., measures the particle size of composite material powders and 17-4PH metal powders. Jasco V-670 (JASCO Corporation, Tokyo, Japan) was used to measure the laser reflectivity of 17-4PH metal powder and composite material powder to determine whether they can absorb lasers of specific wavelengths to ensure manufacturability. A differential scanning calorimeter (DSC) was used to observe the melting point of the adhesive during heating, as a reference for setting the preheating temperature during 3D printing. At the same time, thermogravimetric analysis (TGA) on the adhesive can be used as a basis for temperature retention during the degreasing stage of high-temperature sintering processes. Scanning Electronic Microscopy (JEOL JSM-6390LV) from the United States was used to observe whether the adhesive was successfully wrapped around the 17-4PH powder and to observe the deformation of the object during printing, as well as to compare the conditions after printing, degreasing, and sintering.

The removal of adhesive is carried out in the degreasing process and high-temperature sintering process. The degreasing process uses the SinterZone STZ-C200 oxalic acid catalytic degreasing furnace (represented by Deer Country Technology Co., Ltd., Guangdong, China). The consumption of anhydrous oxalic acid (Uranus Chemicals Co., Ltd., Hsinchu County, Taiwan) is 5 g/min, the nitrogen consumption is 50 L/min, the degreasing temperature is 140 °C, the degreasing time is 5 h, and the thickness of the degreased object is 5 mm. The sintering process uses a Hiper BJ-200GR vacuum debinding sintering furnace (represented by DeerCountry Technology CO., Ltd. Taipei City, Taiwan (R.O.C)), and the debinding sintering process setting during the sintering process is based on a thermogravimetric analysis (TGA) analyzer. The defatted object is kept at 1310 °C for 180 min, and the heating rate of the high-temperature sintering section is 2.5 °C/min. The green body of the paper represents the 3D-printed state, the brown body represents the degreased state, and the silver body represents the high-temperature sintered state.

## 3. Results and Discussion

### 3.1. Material Characterization

**Figure 4:**
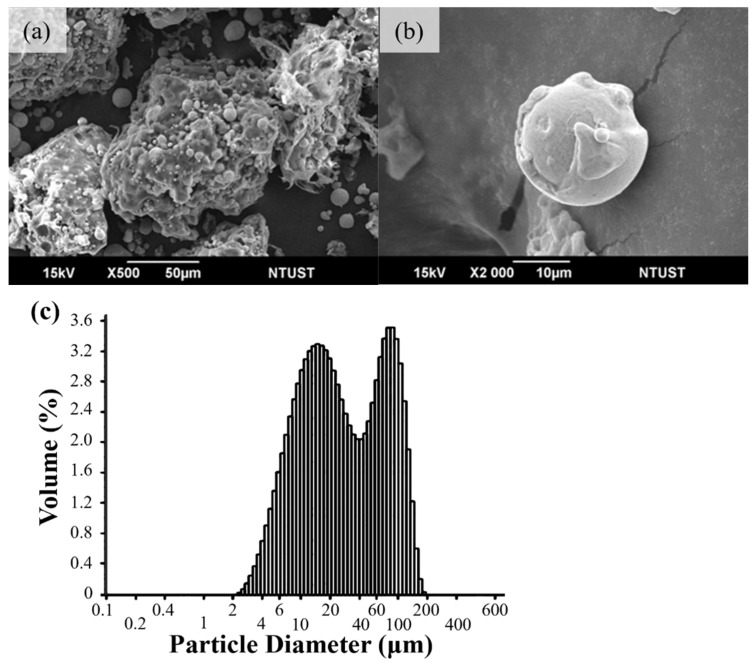
(**a**) Morphology of composite powder core–shell, (**b**) structure diagram of composite powder core–shell, and (**c**) powder size distribution of composite powder.

The SEM image in Figure 4 shows (a) the morphology of the composite powder core–shell, (b) the structure of the composite powder core–shell, and (c) the particle size analysis of the composite powder. The adhesive with a low melting point is mechanically stirred and heated together with 17-4PH powder. During the process, the adhesive melts and becomes viscous. With the adsorption effect of the material capillary phenomenon, 17-4PH powder is bonded into clusters. Figure 4a shows the composite powder that has been bonded into clusters and powdered, with obvious clustering observed. Figure 4b shows the scattered composite powder, with the core 17-4PH powder particles and the surrounding binder, exhibiting a typical core–shell structure. Figure 4c shows the PSD calculation results of the composite powder after sieving, with a D50 of 24.81 μm.

**Figure 5:**
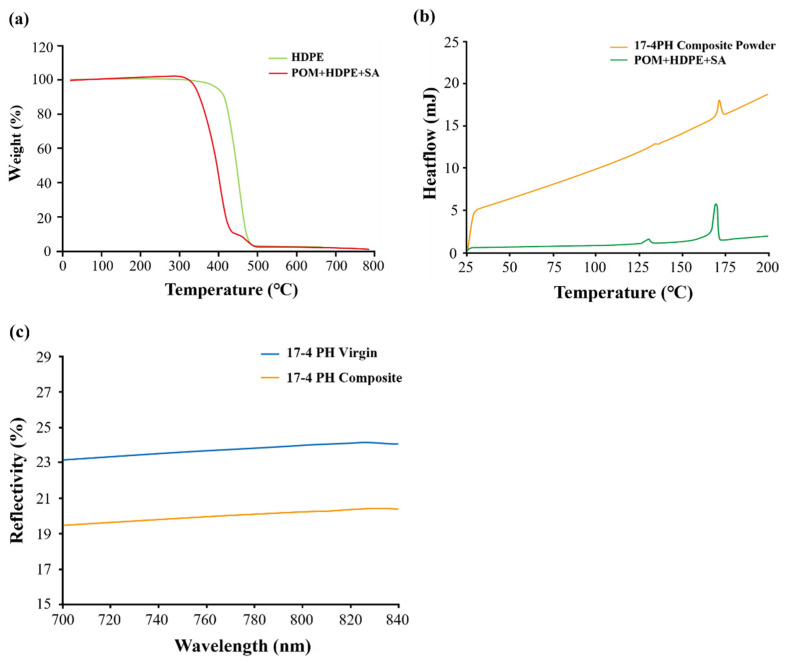
(**a**) TGA of adhesive and HDPE, (**b**) DSC of adhesive and composite material, and (**c**) UV-VIS of composite powder.

Figure 5 shows TGA to test the cracking state of the bonding agent and HDPE during the heating process. The sample is heated from room temperature (25 °C) to 800 °C at a heating rate of 20 °C/min under a nitrogen atmosphere. The TGA results showed that the adhesive began to lose weight at 285 °C, and almost no weight was reduced near 500 °C. The temperature at which the weight changed significantly was between 339 °C and 420 °C. HDPE began to experience weight loss at 330 °C, but there was no significant weight change near 500 °C. There was significant weight loss between 412 °C and 475 °C. According to the detection data, the subsequent DSC detection temperature should be set below 285 °C to avoid the risk of material cracking. At the same time, the reference for setting the sintering process curve can also be obtained.

Figure 5b shows DSC analysis, expressing the changes in the adhesive during heating. DSC detection reveals the thermal changes in the material during the heating process. According to the TGA test results in Figure 5a, it is known that the adhesive begins to lose weight at 285 °C, and the heating temperature for DSC testing should be below 285 °C. Heating of the material at a rate of 10 °C/min is analyzed. The DSC results indicate that the composite powder exhibits two melting peaks. The first melting peak appears between 123 °C and 136 °C, corresponding to HDPE, while the second melting peak appears between 159 °C and 175 °C, corresponding to POM. This message indicates that the maximum temperature for producing composite materials of 17-4PH powder and binder should be between 159 °C and 175 °C. At the same time, we also learned about the preheating temperature range during the printing process. Two key data points were obtained through DSC: the manufacturing material and the preheating temperature for printing.

Figure 5c shows the spectrum analyzer displaying the 17-4 pH composite powder tested using the spectrometer. After mixing, the reflectivity of the composite powder was also measured for comparison. The results showed that the near-infrared spectral reflectance of the composite powder at 808 nm wavelength was 20.27%, which was 3.73% lower than the original powder’s reflectance of 24.00%. This phenomenon can be explained by the fact that after mixing, the adhesive wraps around the surface of the powder, forming a semitransparent surface, resulting in a decrease in reflectivity.

### 3.2. Printing Parameters

**Figure 6:**
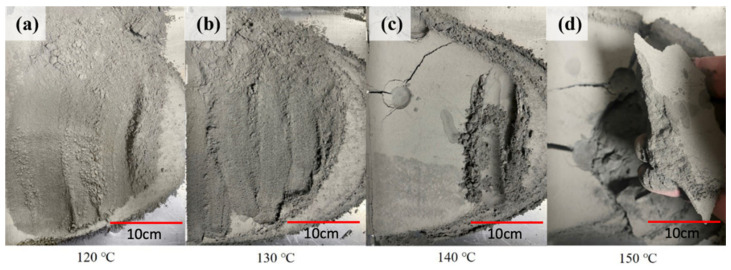
17-4 PH composite powder preheated at four different temperatures (**a**) 120 °C, (**b**) 130 °C, (**c**) 140 °C and (**d**) 150 °C (from left to right).

Figure 6 shows the print preheating test conducted. According to the DSC data (Figure 5b), it can be seen that the starting temperature of the first melting peak of the composite powder is 120 °C. Therefore, Figure 6 shows that the preheating temperatures tested in the experiment were 120, 130, 140, and 150 °C. The results showed that the printing slot remained in powder form at preheating temperatures of 120 °C and 130 °C. At a preheating temperature of 140 °C, it looks like a complete block. A gentle poke at the groove will cause it to break, and even after a light rub, it remains in powder form. At 150 °C, the entire printing slot clumps together. Therefore, the preheating temperatures selected for this experiment are 120 °C, 130 °C, and 140 °C. The selected laser energy densities are 19.28, 35.87, and 52.46 mJ/mm^2^.

**Figure 7:**
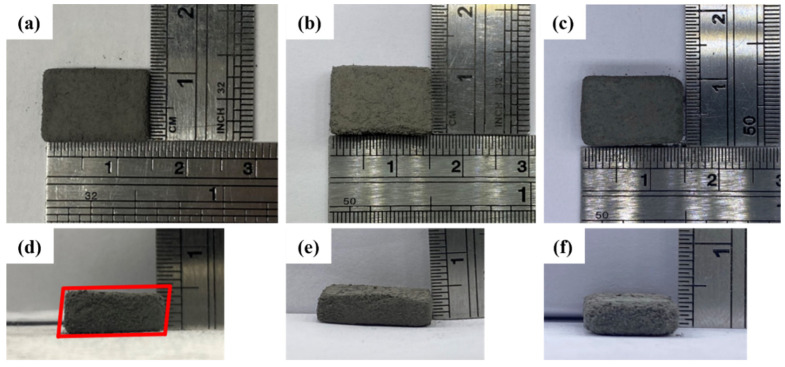
Effects of printing with different layer thicknesses: (**a**,**d**) 75 μm layer thickness (red box shows a parallelogram shape on the side with a thickness of 75 μm); (**b**,**e**) 100 μm layer thickness; and (**c**,**f**) 125 μm layer thickness.

Figure 7 shows the effect of layer thickness on the green body. The forming state of the green body is closely related to the layer thickness, mainly depending on the forming energy. When the energy cannot form a fixed thickness, the object will remain in powder or sheet form. This study observes the effect of different thicknesses on forming to ensure shape integrity before continuing the degreasing process. Figure 7a,d show a parallelogram shape on the side with a thickness of 75 μm. Figure 7b,e show the minimum deformation at a thickness of 100 μm. Figure 7c,f show that the thickness is 125 μm, and the corners are rounded and loose. The printing thickness is set to 100 μm.

**Figure 8:**
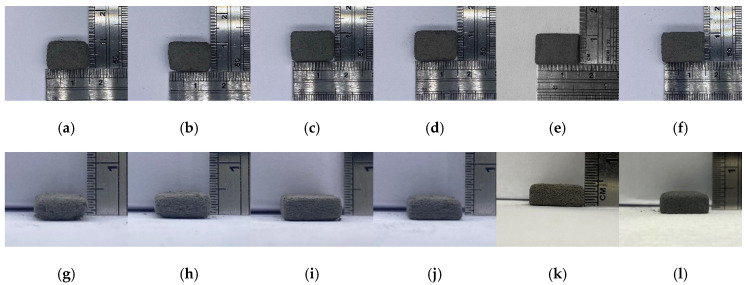
The laser printing temperature and laser energy density experiment: (**a**,**g**) 130 °C, 19.28 mJ/mm^2^; (**b**,**h**) 130 °C, 35.87 mJ/mm^2^; (**c**,**i**) 130 °C, 52.46 mJ/mm^2^; (**d**,**j**) 140 °C, 19.28 mJ/mm^2^; (**e**,**k**)140 °C, 35.87 mJ/mm^2^; (**f**,**l**) 140 °C, 52.46 mJ/mm^2^.

Figure 8a–l show the density and size of green bodies printed using different laser energy densities at preheating temperatures of 130 °C and 140 °C (Table 2). It can be observed that changes in laser energy density affect the growth of green bodies, transitioning from loose structures to stable forms, with corners gradually becoming sharper. At a preheating temperature of 140 °C, all three laser energy densities can produce high-intensity green bodies. The shape is complete and exhibits the highest green density, but excessive energy not only causes deformation but also melts and binds the surrounding powder to the object, resulting in a larger green body size and heavier weight. In this experiment, with a preheating temperature of 140 °C and a laser energy density of 35.87 mJ/mm^2^, the maximum green body density achieved was 3.61 g/cm^3^, and there was an increase of 20.33% compared to the minimum green body density of 3.00 g/cm^3^. Therefore, the subsequent degreasing and sintering experiments will use a layer thickness of 100 μm, a preheating temperature of 140 °C, and a laser energy density of 35.87 mJ/mm^2^ to print the samples.

### 3.3. Debinding and Sintering Parameters

**Figure 9:**
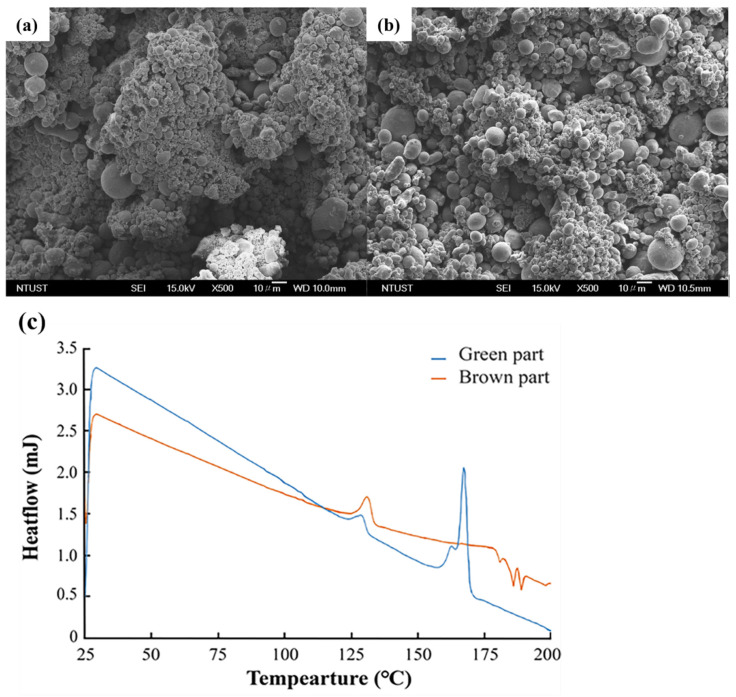
(**a**) DSC results before degreasing, (**b**) after degreasing, and (**c**) DSC results before and after degreasing.

Figure 9c shows the DSC test results of the printed green body and degreased brown body against HDPE and POM in the adhesive. HDPE and POM have different melting peaks. Based on the principle of degreasing, the degreasing temperature is only 140 °C, much lower than the decomposition temperature of HDPE at 330 °C. The degreasing process only removes POM material. DSC can be used to test the melting point and phase transition temperature of materials, as well as to identify different types of materials. To confirm the degreasing process, this study used DSC to detect the endothermic state of green and brown bodies during heating and compared their respective DSC curves. However, the DSC curve of the brown body only showed a clear HDPE melting peak, and compared with the DSC curve of the green body, there was no obvious curve between 140 °C and 175 °C, indicating that only POM material was basically removed, while HDPE was not significantly removed. Figure 9a shows the bonding phenomenon of composite materials after laser sintering. Figure 9b shows the phenomenon of POM reduction after green body degreasing. It was observed that the green body exposed some particles of 17-4PH powder due to POM reduction and also bonded into clusters. According to the DSC test results in Figure 9c, HDPE was the main component of bonding.

**Figure 10:**
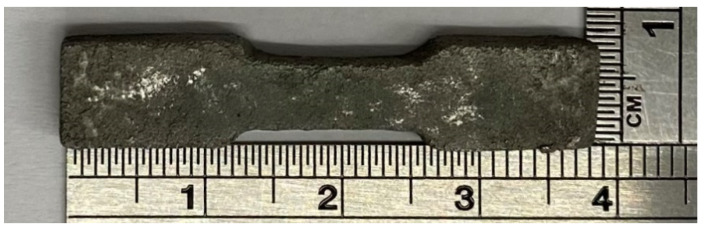
Sintered dog bone sample.

Table 3 shows the mechanical strength of the sintered brown body. According to TGA test results, HDPE begins to experience weight loss at 330 °C, with no significant weight change around 500 °C, and significant weight loss between 412 °C and 475 °C. Therefore, the thermal desorption stage of sintering is set to stay at 350 °C and 450 °C for 120 min, respectively. Nitrogen gas is introduced throughout the thermal desorption stage at a flow rate of 25 L/min. The highest temperature is set at 1310 °C for 180 min, and the total sintering time is 22 h. The final dog bone sample obtained is shown in Figure 10. It was found that the average shrinkage rate after sintering is 22%, the density is 7.57 g/cm^3^, reaching a theoretical density of 97%, the hardness is HRC 14.8, reaching 57% of the theoretical value, and the tensile strength is 605.64 MPa, reaching 65% of the theoretical value.

**Figure 11:**
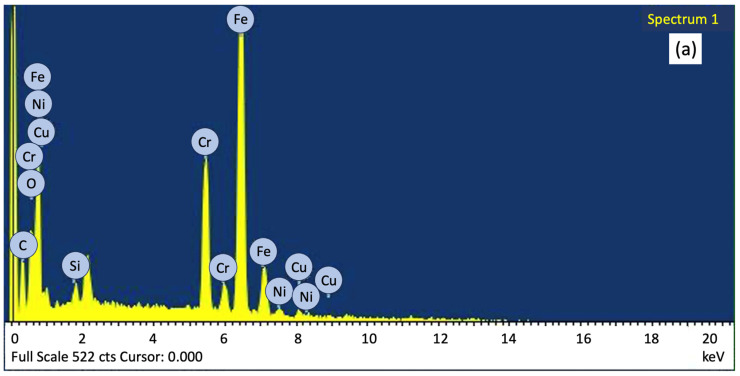

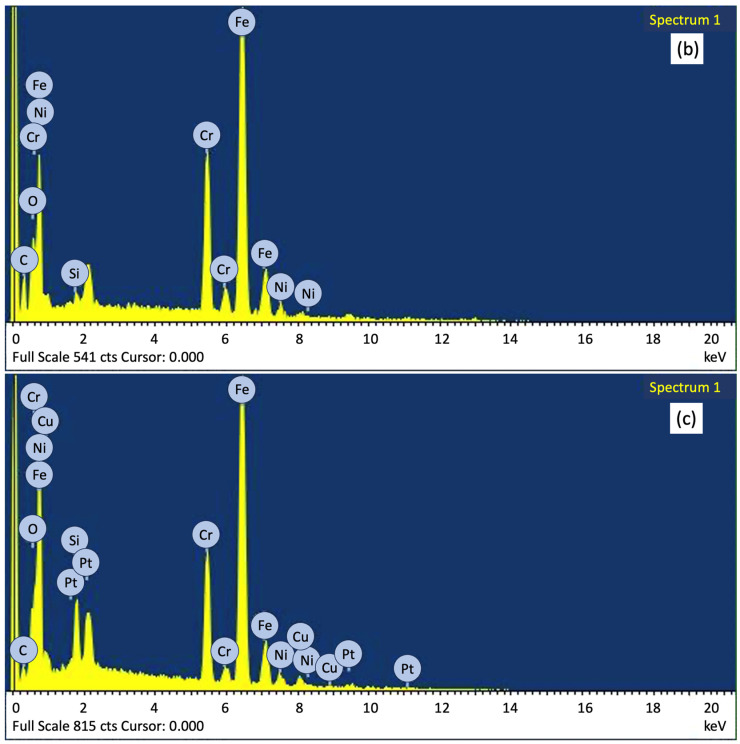
(**a**) Elemental analysis of green billets, (**b**) elemental analysis of brown billets, and (**c**) elemental analysis of silver billets.

Figure 11a–c show the differences in carbon content at different stages, ranging from 8.24 wt% carbon content in the green blank after printing to 5.32 wt% carbon content in the brown blank after degreasing, with 2.64 wt% carbon content remaining in the silver blank after sintering. The oxygen content also increased from 3.45 wt% for green billets to 2.31 wt% for silver billets.

It is known that the chemical formula of POM (polyoxymethylene) is (CH_2_O)_n_, the chemical formula of HDPE (high-density polyethylene) is (C_2_H_4_)_n_, and the chemical formula of SA (stearic acid) is C_18_H_36_O_2_. Therefore, EDS was used to observe the changes in carbon and oxygen elements to confirm the state of the reduced binder, as shown in Table 4. From the degreasing stage, the binder was lost, and after sintering, it was almost removed.

### 3.4. Microscopic Observation and Micro-CT Scan

**Figure 12:**
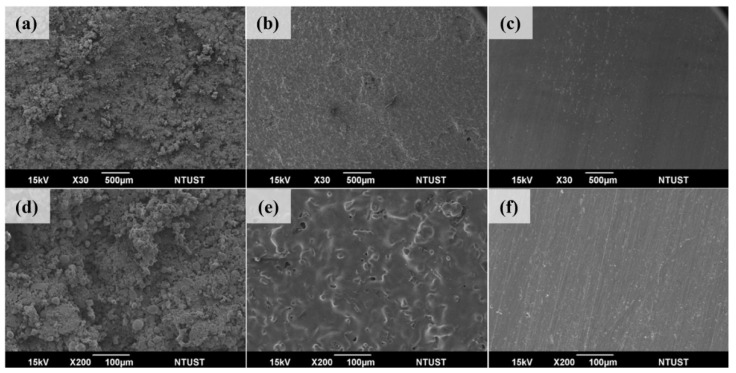
The front of the object (**a**,**d**) is printed, (**b**,**e**) sintered, and (**c**,**f**) ground after sintering.

Figure 12 shows the front of the sample, including after printing, sintering, and grinding after sintering. Printing was carried out using the optimal parameters of a layer thickness of 100 μm, a preheating temperature of 140 °C, and a laser energy density of 52.46 mJ/mm^2^. The degreasing parameters were as follows: anhydrous oxalic acid consumption of 5 g/min, nitrogen consumption of 50 L/min, degreasing temperature of 140 °C, and degreasing time of 5 h. Finally, the brown body was metalized by high-temperature sintering. Figure 12a,d show the characteristics of the adhesive bonding into clusters in composite materials due to laser sintering. There are many metal powders in the material, making the shape of the sintered neck complex and not obvious. Figure 12b,e show the surface after sintering, in a no powder state, with a good sintering state of metal powder, and visible large pores. Figure 12c,f show the surface after grinding. To confirm whether only the surface has large pores after sintering, removing the rough layer on the surface showed that there were no large pores after grinding, only some small pores.

**Figure 13:**
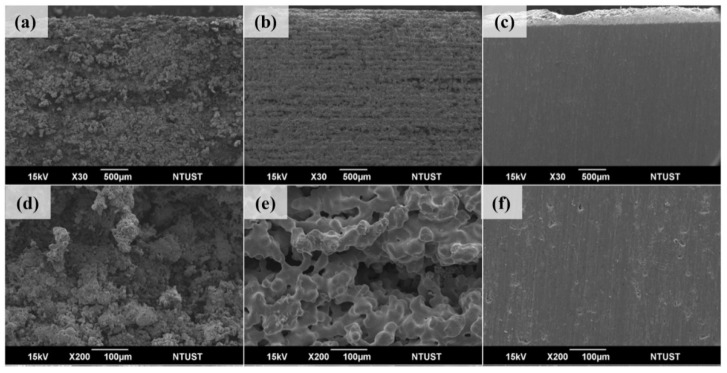
The side of the object (**a**,**d**) after printing, (**b**,**e**) after sintering, and (**c**,**f**) after sintering and grinding.

Figure 13 shows the side of the object, including after printing, sintering, and grinding after sintering. When printing, the adhesion between layers can affect the strength of the green body and also affect the density after high-temperature sintering. Figure 13a,d show the side features after laser sintering. Although there is bonding between layers, there is a clear layer-to-layer structure and a complex sintered neck. Figure 13b,e show the side profile after sintering. There are obvious shrinkage layers and layer structures after sintering, and some irregular and deep pores can also be seen. Figure 13c,f show the side surface after grinding. No larger pores were observed, but after high-temperature sintering, the shrinkage was good. There were some small pores, which were larger and deeper compared to the plane.

**Figure 14:**
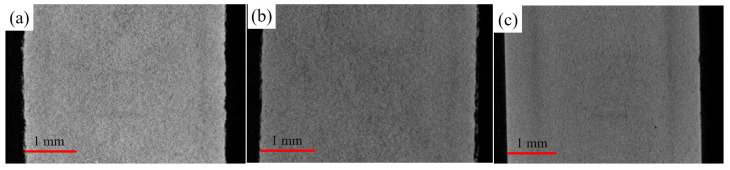
Micro-CT scan of porosity distribution inside and on the surface during (**a**) printing, (**b**) degreasing, and (**c**) sintering.

Figure 14 shows the micro-computed tomography scanner in various states, using BRUKER SKYSCAN 1276 (Billerica, MA, USA). The differences between the surface and interior of green, brown, and silver bodies were compared. Figure 14a shows the internal and surface states of the green body. The interior is filled with adhesive and appears white, with obvious irregularities on the surface. Figure 14b shows the internal and surface states of the brown body. After degreasing, POM is removed, leaving behind HDPE, which shows a darker color inside and more irregularities on the surface. Figure 14c shows the internal and surface states of the silver body. After high-temperature sintering, the density reaches 97% of the theoretical value, the interior becomes finer, and the surface is smooth and straight.

### 3.5. Heat Treatment and Application

**Figure 15:**
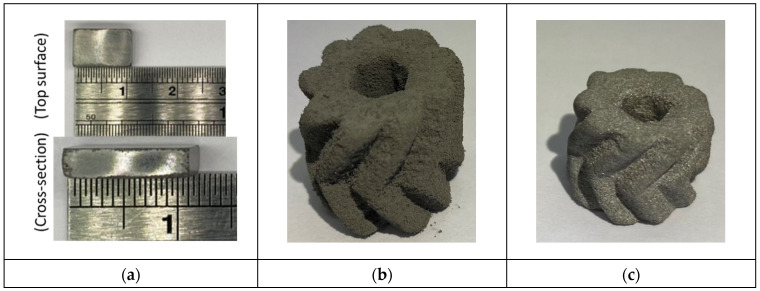
(**a**) As-sintered bulk, (**b**) brown body, and (**c**) sintered specimen.

Table 5 shows the H900 aging treatment of the sintered tensile samples. The tensile strength increased from 605.64 MPa to 931.89 MPa, an increase of 53.87%. In terms of hardness, it increased from HRC 14.80 to HRC 30.76, an increase of 107.84%. Figure 14a shows the (dimensions) appearance of the sintered final block part from the top view and cross-sectional view. Figure 15b,c show the additive manufacturing method applied to nine-tooth helical gears. Traditionally, helical gears are produced through mold casting and CNC milling, which takes a considerable amount of time or results in significant material waste. The method developed in this study can effectively and flexibly produce double helical gears.

## 4. Process Comparison

In comparison with the other powder bed processes such as the SLM and BJT, the advantages of using core–shell powder with SLS technology are listed in Table 6. The SLS process stands out in several areas:

1. Materials and particle size: The core–shell powder technology provides more versatility in material choices than SLS and BJT processes. SLS has a finer powder particle size (D90 < 22 µm), offering more precision and potentially higher resolution compared to SLM, which has a larger particle size (16–63 µm). BJT also uses a similar powder size to SLS, but the precision and application may vary based on the material and binder interaction. The SLS process in this study can use atomized powder or lower-cost water-atomized powder.
2. Mechanical properties: While SLM provides the highest tensile strength (1310 MPa) and sintered density (99.87%), SLS is more advantageous in terms of flexibility with different material types and ease of post-processing. With a tensile strength of 931.89 MPa and a sintered density of 97.3%, SLS offers a good balance of material properties for applications requiring plastic and composite materials.
3. Post-processing: SLS benefits from an acid catalysis post-processing method, which is less labor-intensive than BJT’s hot degreasing and more effective than SLM’s lack of post-processing.

In conclusion, SLS stands out for its versatility in material usage, ease of post-processing, and precision with fine particle sizes, making it a better choice than SLM and BJT for applications involving plastics and composites. While SLM excels in mechanical properties for metal parts, SLS is more adaptable for a broader range of industries and materials. By utilizing the core–shell composite material, it can expand its application and maximize its utility.

## 5. Conclusions

The present study demonstrated the process of mixing 17-4 PH stainless steel powder with a polymer-based binder to successfully develop a composite metal powder with low reflectivity. The adhesive coating method in this paper consists of two steps: step one is material weighing, and step two is heating and stirring the metal powder and adhesive at a specific temperature until they form a cluster, at which point the adhesive is coated on the outside of the powder. When sintering at 140 °C with a laser power of 35.87 mJ/mm^2^ and a layer thickness of 100 μm, a dense and complete shape was achieved with a density of 3.61 g/cm^3^. After degreasing and sintering at 1310 °C for 180 min, the specimen exhibited significant shrinkage (22% across all axes), and a density of 7.57 g/cm^3^ (97% of theoretical density). The mechanical properties such as a hardness of HRC 14.8 (57% of theoretical hardness), with a tensile strength of 605.64 MPa (65% of theoretical value) were achieved for the sintered samples. Notably, after heat treatment, the specimen showed a remarkable improvement, with tensile strength increasing by 53.87% to 931.89 MPa and hardness rising by 107.84% to HRC 30.76, indicating substantial material enhancement. These results highlight the effectiveness of the low-power laser sintering and heat treatment processes in improving the mechanical properties of 17-4 PH stainless steel composites.

## Figures and Tables

**Table 1:** Properties of the adhesive materials.

Binder Role	Component	Brand	Model	Density (g/cm^3^)	Melting Point (°C)
Filler	POM	Asahi Kasei	9520	1.41	165~175
Skeleton	HDPE	Formosa Plastics	8230	0.952	131
Interface activity	SA	First Chemical	First Chemical	0.9408	69.3

**Table 2:** The laser printing temperature and laser energy density.

No.	Preheating Temperature (°C)	Laser Fluence (mJ/mm^2^)	Weight (g)	Density (g/cm^3^)
A	130	19.28	1.33	3.00
B		35.87	1.62	3.16
C		52.46	1.72	3.20
D	140	19.28	1.83	3.27
E		35.87	1.89	3.61
F		52.46	2.14	3.56

**Table 3:** Mechanical strength of brown body after sintering.

	Density (g/cm^3^)	Hardness (HRC)	Tensile Strength (MPa)
Test value	7.57	14.8	605.64
Theoretical value	7.8	26	932
Proportion (%)	97	57	65

**Table 4:** Comparison of carbon and oxygen content of green, brown, and silver billets.

	Green Part	Brown Part	Silver Part
Carbon content	8.24 wt%	5.32 wt%	2.64 wt%
Oxygen content	3.45 wt%	2.90 wt%	2.31 wt%

**Table 5:** Mechanical properties of heat treatment.

	Before Heat Treatment	After Heat Treatment	Relative Value (%)
Tensile Strength (MPa)	605.64	931.89	53.87%
Hardness (HRC)	14.8	30.76	107.84%

**Table 6:** Process comparison of SLS and BJT processes.

		SLM [15]	SLS (This Study)	BJT [16]
Equipment	Forming method	Laser	Laser	Adhesive and light exposure
	Heating module	One	One	Two
Material	Printing materials	Metal powder	Plastic and composite materials	Inorganic powder and binder
	Powder type	Spherical shape	Unlimited	Spherical shape
	Particle size range	16~63 µm	D90 < 22 µm	D90 < 22 µm
Post-processing	Degreasing method	None	Acid catalysis	Hot degreasing
Mechanical properties (H900)	Tensile strength	1310 MPa	931.89 MPa	1275 MPa
	Hardness (HRC)	41	30.76	41.9
	Sintered density	99.87%	97.3%	97.69%

## Data Availability

The original contributions presented in this study are included in the article. Further inquiries can be directed to the corresponding author.

## References

[1] Molotnikov A., Kingsbury A., Brandt M. (2021). Current State and Future Trends in Laser Powder Bed Fusion Technology. Fundamentals of Laser Powder Bed Fusion of Metals.

[2] Singh R., Gupta A., Tripathi O., Srivastava S., Singh B., Awasthi A., Rajput S.K., Sonia P., Singhal P., Saxena K.K. (2020). Powder Bed Fusion Process in Additive Manufacturing: An Overview. Mater. Today Proc..

[3] Chen A.N., Wu J.M., Liu K., Chen J.Y., Xiao H., Chen P., Li C.H., Shi Y.S. (2018). High-Performance Ceramic Parts with Complex Shape Prepared by Selective Laser Sintering: A Review. Adv. Appl. Ceram..

[4] Bhat C., Prajapati M.J., Kumar A., Jeng J.Y. (2024). Additive Manufacturing-Enabled Advanced Design and Process Strategies for Multi-Functional Lattice Structures. Materials.

[5] Tiwari S.K., Pande S., Agrawal S., Bobade S.M. (2015). Selection of Selective Laser Sintering Materials for Different Applications. Rapid Prototyp. J..

[6] Schmid M., Amado A., Wegener K. (2015). Polymer Powders for Selective Laser Sintering (SLS). Proceedings of the AIP Conference Proceedings.

[7] Kruth J.P., Wang X., Laoui T., Froyen L. (2003). Lasers and Materials in Selective Laser Sintering. Assem. Autom..

[8] Singh S., Sharma V.S., Sachdeva A. (2016). Progress in Selective Laser Sintering Using Metallic Powders: A Review. Mater. Sci. Technol..

[9] Anand M., Das A.K. (2021). Issues in Fabrication of 3D Components through DMLS Technique: A Review. Opt. Laser Technol..

[10] Yan C.Z., Shi Y.S., Yang J.S., Liu J.H. (2009). Preparation and Selective Laser Sintering of Nylon-Coated Metal Powders for the Indirect SLS Process. Rapid Prototyp. J..

[11] Li M., Du W., Elwany A., Pei Z., Ma C. (2020). Metal Binder Jetting Additive Manufacturing: A Literature Review. J. Manuf. Sci. Eng. Trans. ASME.

[12] Kutlu Y., Wencke Y.L., Luinstra G.A., Esen C., Ostendorf A. (2020). Directed Energy Deposition of PA12 Carbon Nanotube Composite Powder Using a Fiber Laser. Procedia CIRP.

[13] Du W., Ren X., Pei Z., Ma C. (2020). Ceramic Binder Jetting Additive Manufacturing: A Literature Review on Density. J. Manuf. Sci. Eng. Trans. ASME.

[14] Chao Y., Liu S., Yeh D., Kumar A., Tsai J., Prajapati M.J., Jeng J. (2024). Development of Carbon Black Coating on TPU Elastic Powder for Selective Laser Sintering. Materials.

[15] Material Data Sheet—FlexLine EOS StainlessSteel 17-4PH. https://wipro-3d.com/pdf/SS-17-4-PH.pdf.

[16] Desktop Metal—17-4PH Stainless Steel. https://www.desktopmetal.com/resources/174-stainless-steel.

